# Understanding Ionic Transport in LiFeO_2_ Polymorphs: Pathways to Enhancing Lithium-Ion Battery Performance

**DOI:** 10.3390/ma19163378

**Published:** 2026-08-08

**Authors:** João R. Da Fonseca, Borja Caja-Muñoz, María E. Dávila, Juan P. Martínez-Pastor, Juan F. Sánchez-Royo, Maria C. Asensio

**Affiliations:** 1 Institut de Ciència dels Materials de la Universitat de València (ICMUV), University of Valencia, Carrer del Catedrátic José Beltrán Martinez, 2, 46980 Paterna, Spain; 2 GREENER Group, Institute of Materials Science of Madrid (ICMM), Spanish National Research Council (CSIC), 28049 Madrid, Spain; 3 MATINÉE: CSIC Associated Unit Between ICMM and ICMUV, 28049 Madrid, Spain

**Keywords:** CAVD, BVSE, ionic migration, LiFeO_2_, energy storage

## Abstract

We compare the ionic transport properties of six LiFeO_2_ polymorphs side by side to assess their potential as intercalation cathodes for lithium-ion batteries (LIBs). Employing two established methods, Bond Valence Site Energy (BVSE) calculations and Crystal Analysis by Voronoi Decomposition (CAVD), with identical settings for all six structures, we analyze how structural variations affect Li-ion diffusion pathways, dimensionalities, and migration barriers. Within this comparison, the ordered rock salt phase shows the most favorable transport characteristics, combining the lowest energy barriers of the six polymorphs with three-dimensional Li-ion conduction. The tetrahedral polymorph also has a three-dimensional void network, but its lowest-energy migration is one-dimensional, and the network connects in all three dimensions only at considerably higher energy. In contrast, layered and corrugated structures enable quasi-two-dimensional diffusion but are limited by high out-of-plane barriers. The goethite and γ phases are the most constrained. Goethite confines low-energy migration to a single axis, and in γ-LiFeO_2_, high barriers in every direction leave the geometrically connected network kinetically ineffective. Together, the results provide a consistent structure–transport comparison across the six polymorphs that can guide the choice of LiFeO_2_ phases for further cathode development.

## 1. Introduction

Lithium-ion batteries (LIBs) are central to the integration of renewable energy into modern power grids, enabling efficient storage solutions that balance intermittent supply with fluctuating demand. They are also indispensable in portable electronics and electric vehicles, owing to their high energy density, relatively long cycle life, and stable performance under diverse operating conditions [1]. Despite these advantages, conventional LIB technologies still face challenges related to sustainability, cost, and safety. In particular, widely used cathode materials such as LiCoO_2_ raise both economic and environmental concerns due to the scarcity, price volatility, and toxicity of cobalt.

Among the alternative cathode materials investigated, lithium iron oxide (LiFeO_2_) has attracted considerable interest due to its low toxicity, abundance of raw materials, environmental compatibility, and relatively high theoretical capacity of 282 mAhg^−1^ [2,3]. Nevertheless, the practical implementation of LiFeO_2_ has been hindered by intrinsic drawbacks, including sluggish lithium-ion diffusion kinetics, poor electronic conductivity, and difficulties in achieving its full theoretical capacity. These limitations contribute to capacity fading and restrict its widespread application in next-generation LIBs [1,4,5].

To address these issues, a deeper understanding of the ionic transport mechanisms in LiFeO_2_ is required. While ten distinct polymorphs of LiFeO_2_ have been reported (Table 1), with some exhibiting promising electrochemical activity [2,3,6], research has been disproportionately concentrated on the α-LiFeO_2_ phase. In this polymorph, recent improvements in electrochemical performance have been largely attributed to reduced particle size and nanoscale engineering, which enhance surface kinetics and shorten Li-ion diffusion pathways [1,7].

However, despite these advances, a comprehensive understanding of how crystal structure influences Li-ion transport across the various LiFeO_2_ polymorphs remains incomplete. In particular, the relationship between polymorphic phase stability, interstitial geometry, and diffusion dimensionality has not been systematically explored. Addressing this knowledge gap is essential for guiding structural design strategies aimed at improving the electrochemical performance of LiFeO_2_-based cathodes and unlocking their potential as sustainable alternatives for high-performance LIBs.

In the present work, we evaluate the ionic-conduction pathways and migration-energy barriers of six LiFeO_2_ polymorphs using a combined Voronoi-decomposition geometric analysis (CAVD) and the Bond-Valence Site-Energy (BVSE) force field to extract approximate minimum-energy paths (MEPs) for Li-ion migration. Density-functional-theory nudged-elastic-band (DFT-NEB) calculations are the first-principles reference for such barriers, but they converge slowly when the initial migration trajectory is poorly guessed, and must be repeated for every hop of a percolating network. They also often underestimate migration barriers by 0.1–0.3 eV because the effects of lattice dynamics are not included [8]. These limitations make comparative screening of several polymorphs impractical.

The empirical BVSE method developed by Adams and co-workers, as implemented in SoftBV, provides a rapid approximation of migration pathways and barriers. In a published benchmark, SoftBV BVSE migration barriers were compared with the literature ’sDFT-NEB data for 57 Li^+^ and 19 Mg^2+^ ionic conductors, with the authors reporting good agreement in general [9]. Although SoftBV does not reach the precision or transferability of DFT, its computational cost is several orders of magnitude lower [9]. This computational efficiency has enabled its integration into high-throughput platforms that have already screened more than 20,000 candidate compounds [10,11,12]. Importantly, BVSE combined with Voronoi-based geometric analysis not only identifies energetically accessible pathways but also captures their dimensionality and connectivity, which are critical descriptors of long-range ionic conductivity.

Direct comparisons with NEB found fully consistent migration paths for NaZr_2_P_3_O_12_ and tetragonal LLZO [12]. For LiScGeO_4_, FP-NEB reproduced the BVSE minimum-energy path, although the migration barrier changed from 0.39 eV in BVSE to approximately 0.16 eV in FP-NEB, which the authors described as semi-quantitative agreement [10]. For three Li_3_PS_4_ polymorphs, Pan et al. reported Li-ion pathways and activation energies consistent with first-principles calculations [13]. These comparisons support using CAVD–BVSE to map migration pathways and compare approximate thresholds, while precise barrier determination remains a task for first-principles calculations. Building on these precedents, our BVSE–Voronoi calculations rapidly uncover the lithium-ion migration pathways in six LiFeO_2_ polymorphs, demonstrating how subtle differences in crystal topology directly influence ionic conductivity.

In the following sections, we apply a combined CAVD–BVSE approach to six LiFeO_2_ polymorphs: layered, corrugated, tetrahedral, goethite, γ, and ordered rock salt. Both methods are established screening tools, and the contribution of this work lies in the consistency of the comparison rather than in the techniques themselves. Because every polymorph is evaluated with the same protocol, the resulting migration pathways, diffusion dimensionalities, and energy barriers can be compared directly across structures. This side-by-side evaluation clarifies the role of crystal topology in governing ionic conductivity and yields structure–property relationships that identify which LiFeO_2_ phases merit closer study as cathode active materials in LIBs.

### 1.1. Crystal Structures of LiFeO_2_ Polymorphs and Electrochemical Activity

#### 1.1.1. Layer and Corrugated Layer Structures

**Figure 1:**
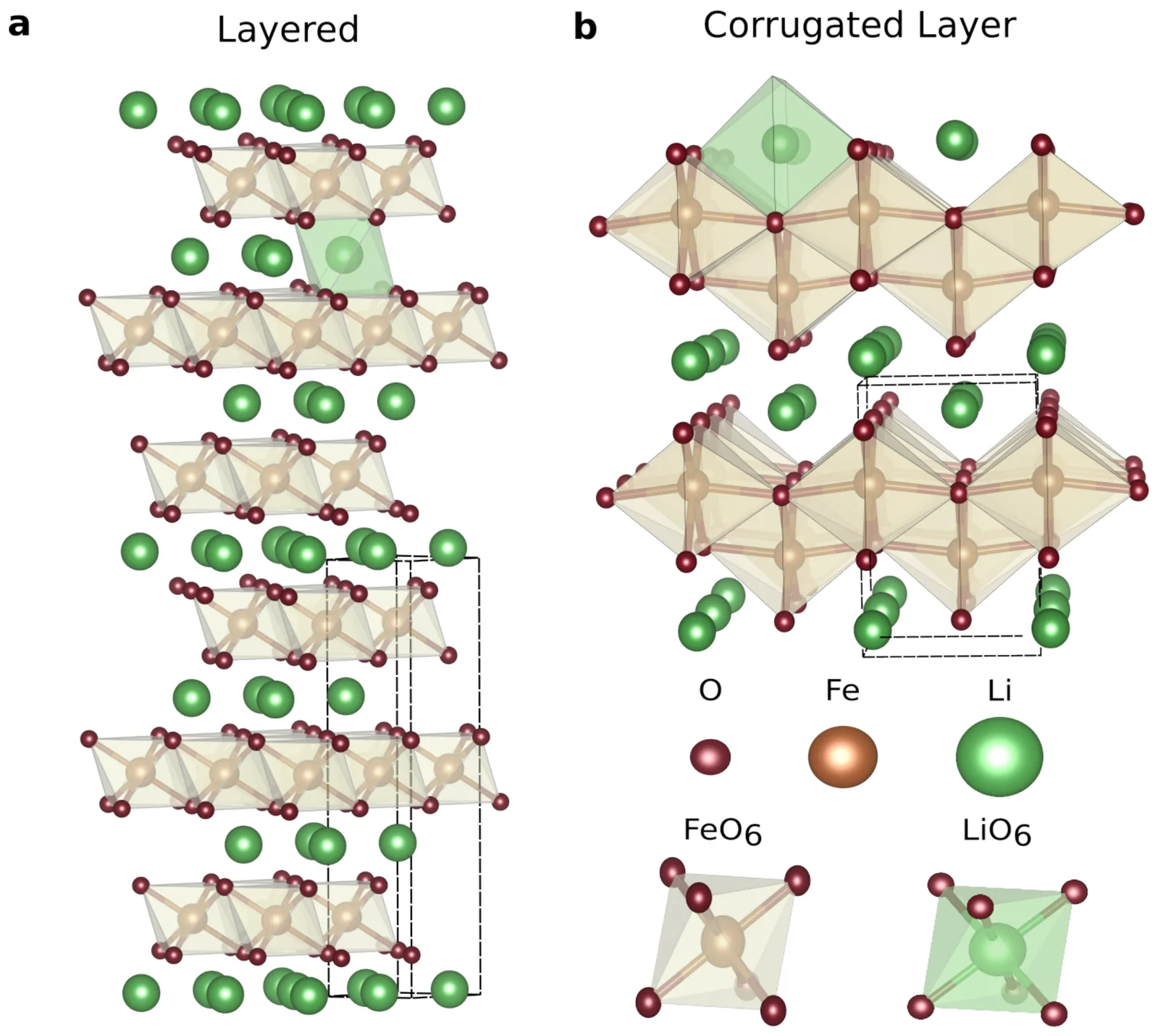
Crystal structures of the (**a**) layered and (**b**) corrugated layer polymorphs of LiFeO_2_.

Figure 1 shows the crystal structures of the layered and corrugated layer polymorphs of LiFeO_2_. The layered polymorph of LiFeO_2_ (Figure 1a) adopts a structure analogous to that of LiCoO_2_, crystallizing in a rhombohedral lattice where cation layers alternate with close-packed oxygen sheets. Lithium and iron ions occupy distinct octahedral sites in successive layers, with the Li layers providing potential diffusion planes parallel to the ab-plane. This arrangement enables quasi-two-dimensional Li-ion transport: diffusion is favored within the interlayer planes, while migration across the layers is strongly hindered by high energy barriers.

The layered polymorph of LiFeO_2_ represents one of the most common structures among ordered lithium metal oxides. The vast majority of commercial lithium-ion batteries employ lithium transition metal layered oxides as cathode materials [14]. This structure is also isostructural with the α-NaFeO_2_ type, classified as O3-LiFeO_2_ according to the Delmas categorization. Typically, the rhombohedral structure comprises alternating layers of edge-sharing FeO_6_ octahedra and lithium layers. In the nomenclature, the “O” indicates that lithium is octahedrally coordinated, while the “3” refers to the number of FeO_6_ sheets within each unit cell [15,16].

In this structure, Li diffusion occurs within the lithium layer, where lithium ions hop between octahedral sites through intermediate tetrahedral sites [17]. Hirayama et al. [15] reported the synthesis of layered LiFeO_2_ containing a secondary ordered rock salt phase, with a mass fraction ratio of 0.637/0.363. The discharge capacity of this material increased from about 90 mAhg^−1^ to 120 mAhg^−1^ over several cycles. During the initial charge, a phase transition to a cubic phase was observed, which may explain the capacity rise. In a subsequent study, presented in Ref. [18], selected-area electron diffraction (SAED) confirmed a transition from the layered phase to an ordered rock salt phase, again accompanied by an improvement in capacity with cycling.

The corrugated layered polymorph (Figure 1b) can be regarded as a distorted variant of the ideal layered phase. In this structure, the cation–oxygen polyhedra are tilted, generating a “wavy” or corrugated stacking of the FeO_6_ and LiO_6_ octahedra. This corrugation lowers the symmetry and produces anisotropic diffusion pathways, where in-plane conduction remains accessible but is constrained by narrower bottlenecks. Out-of-plane transport becomes even less favorable than in the ideal layered phase, further suppressing effective three-dimensional ionic conductivity. This corrugated layer phase of LiFeO_2_ is isostructural with β-NaMnO_2_ and features an orthorhombic structure. Unlike the planar LiO_6_ octahedral layers in the layered phase, the corrugated layer phase consists of zig-zag layers of edge-sharing FeO_6_ octahedra, with lithium ions occupying octahedral sites between the iron oxide layers. Orthorhombic symmetry introduces slight distortions in the layers, causing variations in Fe−O bond lengths and angles that influence the layer spacing and the geometry of the interlayer region [19].

Overall, both layered and corrugated layered polymorphs predominantly support two-dimensional Li-ion transport, with limited percolation pathways along the *c*-axis. The electrochemical properties of the corrugated layer phase for LiFeO_2_ were studied in the late 1990s and early 2000s [20,21,22]. Lee et al. synthesized corrugated LiFeO_2_ containing a minor spinel LiFe_5_O_8_ phase, achieving a discharge capacity of 150 mAhg^−1^ between 4.5 and 1.5 V vs. Li^+^. Capacity retention remained stable until the 13th cycle, after which a 8 % capacity drop occurred, attributed to a complete structural transformation to the spinel phase during cycling. This phase transformation, which is believed to be finalized by the 13th cycle, likely accounts for the observed capacity decline [20].

Later, Lee et al. examined the same compound under a lower current density, observing no capacity drop in the 13th cycle. Instead, discharge capacity increased until the 3rd cycle, with 73% capacity retention after 50 cycles. The spinel phase was found to be fully formed by the 3rd cycle, with minimal phase changes observed between cycles 3 and 50 [22].

#### 1.1.2. Tetrahedral and Goethite Structures

**Figure 2:**
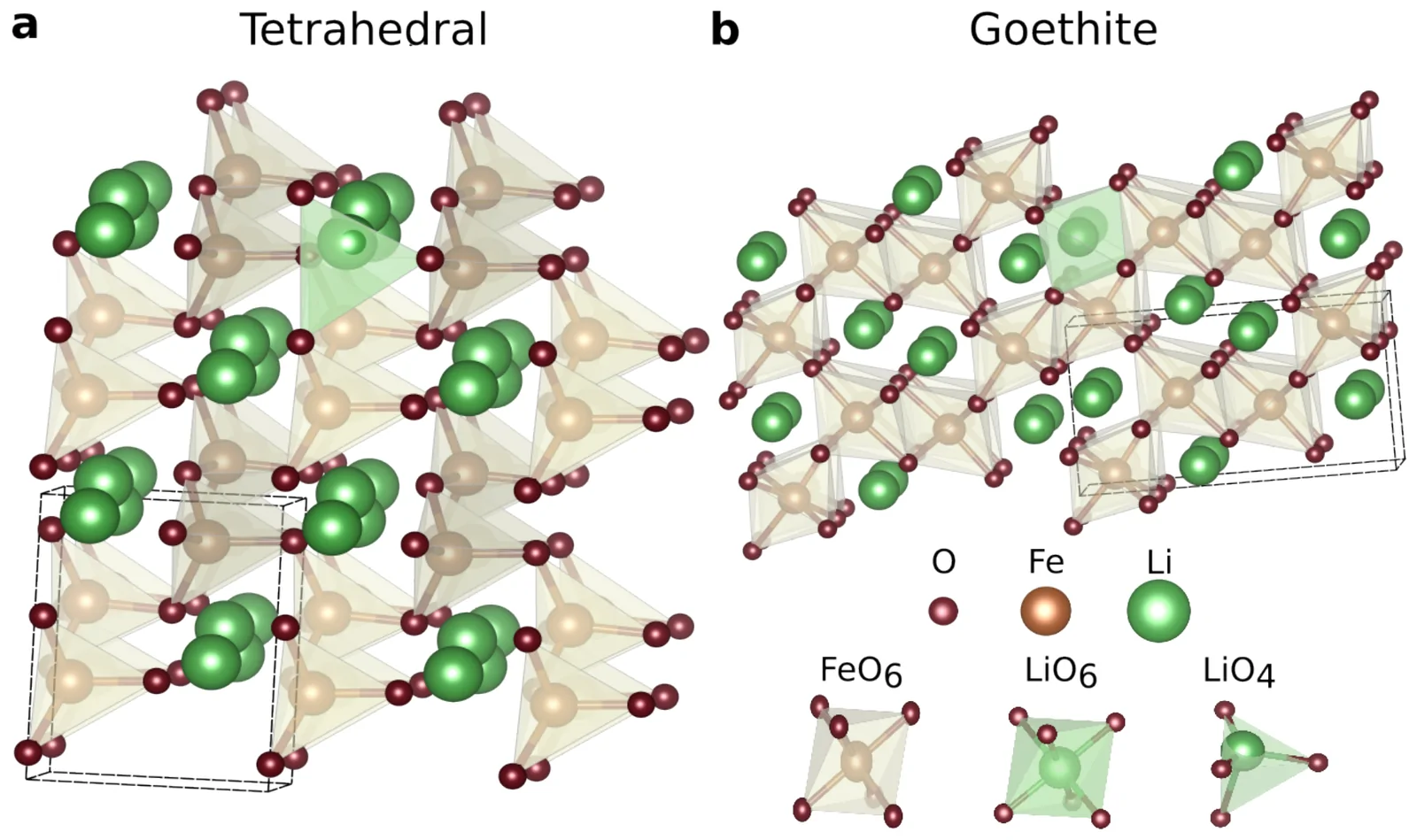
Crystal structures of the (**a**) tetrahedral and (**b**) goethite polymorphs of LiFeO_2_.

While the previously described polymorphs are based on layers or networks of FeO_6_ octahedra, the tetrahedral (*T*-LiFeO_2_) phase represents a distinct structural motif. This structure, shown in Figure 2a, is derived from the orthorhombic β-NaFeO_2_ parent phase and was first reported by Armstrong et al. [23]. In this polymorph, lithium and iron ions occupy half of the available tetrahedral sites in a hexagonal close-packed oxygen framework. The LiO_4_ and FeO_4_ tetrahedra share only corners, forming zigzag rows throughout the lattice. Because of this arrangement, *T*-LiFeO_2_ has been reported to show ionic conductivity comparable to that of olivine-type LiFePO_4_, with Li-ion mobility supported by a geometrically three-dimensional network of interconnected tetrahedral sites.

The connectivity of the tetrahedra defines the diffusion mechanism. Three of the four triangular faces of each LiO_4_ tetrahedron are adjacent to empty octahedral sites. These provide bridges between neighboring tetrahedra, forming characteristic tetrahedron–octahedron–tetrahedron (*t*–*o*–*t*) migration channels. The fourth face is shared with an oppositely oriented tetrahedron (perpendicular to the close-packed planes), which enables Li-ion migration across layers. Once a Li^+^ ion enters such a tetrahedral site, it can readily hop into a neighboring octahedron, thereby accessing interconnected diffusion pathways in all spatial directions. This geometry connects the tetrahedral sites in all three directions in *T*-LiFeO_2_. Whether migration along all three directions is energetically accessible is examined in Section 3.

In contrast, the goethite polymorph of LiFeO_2_, shown in Figure 2b, adopts a markedly different topology. Its structure is composed of edge- and corner-sharing FeO_6_ octahedra forming double chains that run parallel to one crystallographic axis, reminiscent of the mineral FeOOH (goethite). Lithium ions occupy channels formed between these chains, but the connectivity of the diffusion network is far more restricted than in the tetrahedral phase. As a result, Li-ion conduction in the goethite structure is largely one-dimensional and highly anisotropic, with high migration barriers associated with bottlenecks along the chains. Such limited percolation severely hampers long-range ionic transport, which is consistent with the poor electrochemical performance reported for this phase as a cathode active material.

Taken together, the tetrahedral and goethite polymorphs represent two limiting cases of Li-ion transport in LiFeO_2_: the tetrahedral phase offers a relatively open three-dimensional diffusion network, whereas the goethite phase imposes severe structural constraints that strongly impede mobility. This contrast shows how strongly local coordination geometry and framework connectivity control ionic conductivity across the LiFeO_2_ polymorphs.

Regarding electrochemical activity, the ball-milled *T*-LiFeO_2_ electrode was cycled between 1.5 V to 5.1 V at a rate of 100 mAg^−1^, delivering capacities as high as 120 mAhg^−1^. The capacity rose over the first few cycles before stabilizing, with reasonable capacity retention thereafter. On cycling, the structure converts from tetrahedral LiFeO_2_ (distorted hcp packing) to the defective spinel LiFe_5_O_8_ (ccp packing), as confirmed by powder XRD on a cell arrested after 20 cycles.

Few works have been reported describing the synthesis of goethite LiFeO_2_; Sakurai et al. [24] reported one such route. The XRD pattern of the sample was indexed in the orthorhombic system with lattice parameters of a=9.67 Å, b=2.934 Å, and c=5.005 Å; however, the atomic positions were unresolved. Recently, a density functional theory-based method capable of resolving atomic positions was developed [25], and goethite LiFeO_2_ was resolved using goethite FeO(OH) as a prototype. The generated compound is shown in Figure 2b. The FeO_6_ octahedra share edges to form double chains running parallel to the *c* axis. These FeO_6_ octahedra accommodate LiO_6_ octahedra between them. Regarding electrochemical activity, goethite LiFeO_2_ was cycled between 1.5 V to 4.5 V, revealing an initial discharge capacity of 70 mAhg^−1^.

#### 1.1.3. γ-LiFeO_2_ and Ordered Rock Salt Structures

**Figure 3:**
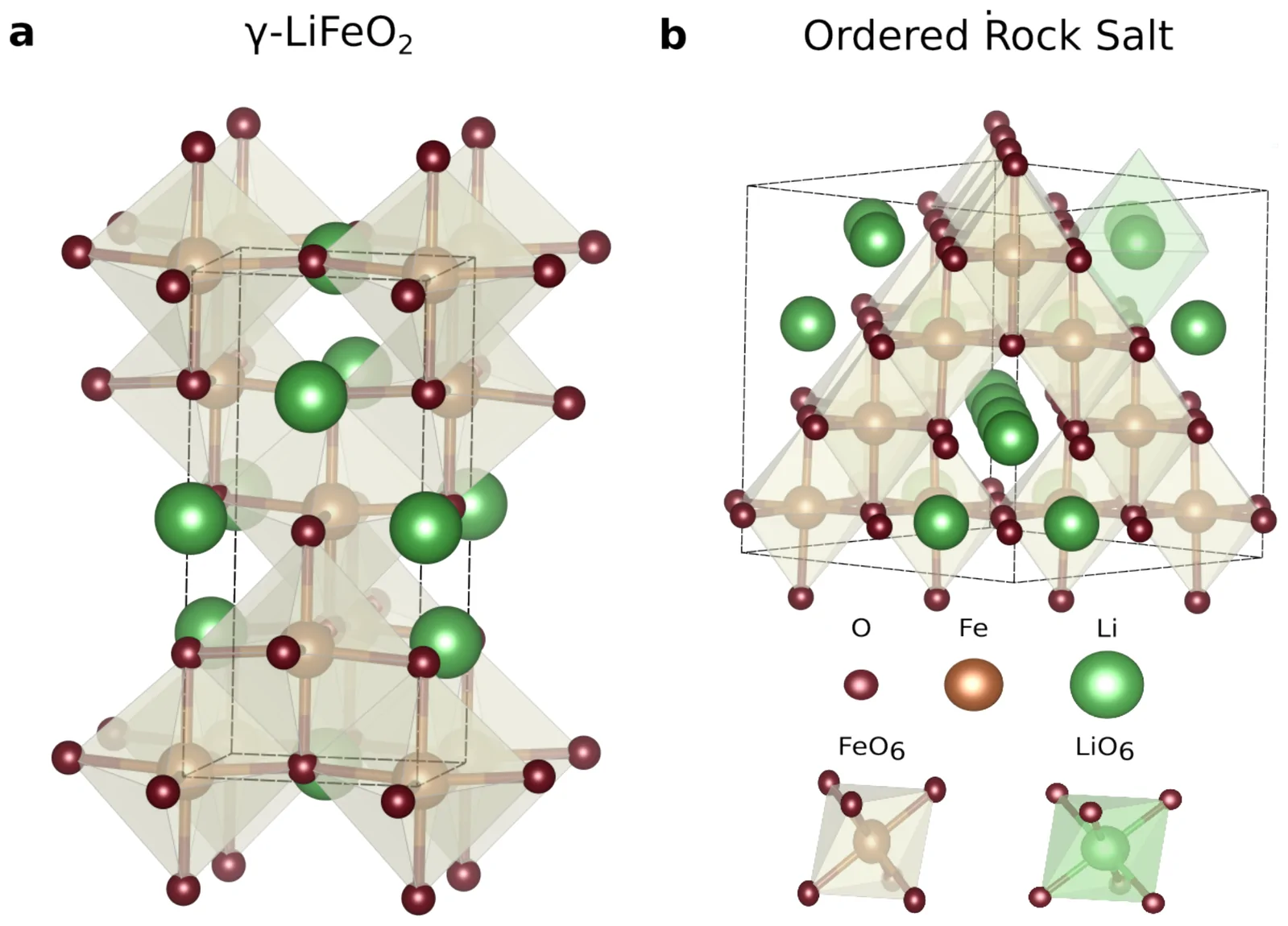
Crystal structures of the (**a**) γ-LiFeO_2_ and (**b**) ordered rock salt polymorphs of LiFeO_2_.

γ-LiFeO_2_ (Figure 3a) is widely recognized as a poor ionic conductor and exhibits limited electrochemical activity [17]. Structurally, it is isostructural with γ-NaFeO_2_, adopting a tetragonal arrangement (space group $I4_{1}/amd$). The framework consists of edge-sharing FeO_6_ octahedra, while lithium ions occupy octahedral sites located between the iron-oxide layers. A key feature of this polymorph is the presence of 2-TM (two-transition-metal) channels, where Li migration would require passage through tetrahedral sites that are coordinated by two Fe cations. These channels are energetically unfavorable, as the activated lithium ions experience strong electrostatic repulsion from the adjacent transition-metal cations, effectively blocking percolating diffusion pathways. Consequently, γ-LiFeO_2_ supports only highly restricted ionic transport and performs poorly as a cathode material in LIBs.

In contrast, the ordered rock-salt polymorph of LiFeO_2_ (Figure 3b), reported in the Materials Project database though not yet synthesized in phase-pure form, displays markedly different characteristics (Section 1.1.1). This cubic phase often emerges as a transient or mixed component within layered LiFeO_2_, and performance improvements observed in such systems are frequently attributed to partial or complete transformation into this ordered rock-salt structure. Its lattice resembles that of the spinel LiMn_2_O_4_, comprising a three-dimensional network of corner-sharing FeO_6_ octahedra interlinked with LiO_4_ tetrahedra. Such connectivity facilitates three-dimensional diffusion pathways with relatively low migration barriers, in stark contrast to the diffusion bottlenecks in γ-LiFeO_2_.

The comparison between γ-LiFeO_2_ and the ordered rock-salt polymorph shows the decisive role of local coordination environment and cation ordering in governing Li-ion transport. Both polymorphs contain topologically connected three-dimensional migration networks, but they differ in whether that connectivity is kinetically accessible. In the tetragonal γ phase, strong electrostatic constraints render the network kinetically ineffective, whereas the cubic ordered rock-salt framework combines the same connectivity with low migration barriers and therefore supports fast Li-ion mobility. This contrast identifies the ordered rock-salt phase as the more favorable of the two for Li-ion transport.

**Table 1:** Structure information of known LiFeO_2_ polymorphs and their reported electrochemical activity.

Type	Space Group	Cryst. Sys.	Structure Type	Order	Capacity (mAh g^−1^)	Ref.
α	Fm-3m	Cubic	NaCl	x	150	[1]
					290.6, 194, and 183.8 ^1^	[7]
β	I4/mmm	Tetragonal	CoO	x	—	[3]
$\beta^{\prime }$	C2/c	Monoclinic	NaErO_2_	x	—	[2,3,26]
γ	I41/amd	Tetragonal	—	✓	1055.3 ^2^	[3]
Layered	R-3m	Rhombohedral	α-NaFeO_2_	✓	115, 210, 390 ^3^	[18]
					100	[15]
					205	[27]
Goethite	Pbnm	Orthorhombic	α-FeOOH	✓	70	[24]
Hollandite	I4/m	Tetragonal	β-FeOOH	x	150	[2]
Corrugated layer	Pmnm	Orthorhombic	β-NaMnO_2_	✓	150	[22]
					150	[21]
					150	[20]
Ordered Rock Salt	Fd-3m	Cubic	LiTiO_2_	✓	—	—
*T*-LiFeO_2_	Pna21	Orthorhombic	β-NaFeO_2_	✓	120	[23]

In the “Order” column, ✓ denotes a phase with a fully ordered cation arrangement, and x one that is cation-disordered, only partially ordered, or for which no ordered model is reported. Only the six phases with a fully ordered structural model (✓) were evaluated in this work. The β/$\beta^{\prime }$ labels follow Refs. [3,26]. Ref. [2] applies β to the monoclinic C2/c phase, and the two labels are often confused in the literature [26]. $\beta^{\prime }$ is only partially ordered [26], and no ordered model is reported for the hollandite phase. ^1^ At 0.1, 1, and 2C, respectively; ^2^ used as anode; ^3^ discharge voltage is 2.0, 1.5, and 1.0 V, respectively.

## 2. Materials and Methods

### 2.1. Bond Valence Site Energy (BVSE) Method

The Bond Valence Site Energy method is a computational approach used to evaluate ion migration pathways and energy barriers in crystalline materials, particularly in solid-state ionic conductors such as battery cathodes, solid electrolytes, and fuel cell materials. It builds upon the bond valence model, which quantifies the strength of bonds between cations and anions in a crystal structure, ensuring local charge balance and structural stability. In BVSE, this concept is extended to generate an energy landscape for mobile ions by calculating the bond valence mismatch at potential migration sites. BVSE provides a semi-quantitative assessment of ion transport properties, including migration barriers, diffusion dimensionality, and percolation pathways. By mapping out site energies across a crystal lattice, it identifies the most favorable conduction routes and helps distinguish between one-dimensional (1D), two-dimensional (2D), and three-dimensional (3D) ion transport mechanisms. This makes it particularly useful for evaluating and predicting the performance of lithium-ion battery materials, where ion mobility is a key factor in determining energy storage efficiency and power density.

Compared to traditional computational methods such as density functional theory (DFT) and molecular dynamics (MD), BVSE offers a computationally efficient alternative that can rapidly screen large numbers of materials. Although it does not explicitly account for electronic interactions or phonon effects, its ability to capture the geometrical and energetic constraints of ionic diffusion makes it a useful tool for material design [28].

The bond valence originates from Pauling’s empirical rules, which state that the bond strength between cations and anions in stable structures should match their valences. This principle initially conflicted with many known compounds but was later resolved by correlating the deviation from Pauling bond strength with bond length. An exponential law was then developed and fitted to multiple cation–anion interactions, making it straightforward to calculate the bond valence sum for any cation–anion pair. To assess whether an ion can migrate within a crystal, one calculates the mismatch between the bond valence sum and the ion’s oxidation state, referred to as the bond valence mismatch. To interpret this mismatch as an energy, Adams et al. [9] developed a force field that computes the potential energy landscape of moving ions on a 3D mesh within the crystal structure, yielding semi-quantitative agreement with DFT and experimental data. In this work, we calculate migration energy barriers and the energy landscape for the six selected polymorphs using CCNB version 0.2.3, which uses CAVD version 0.5.4, developed in Refs. [10,11,13].

### 2.2. Crystal Analysis by Voronoi Decomposition (CAVD) and Joint Method

The Crystal Analysis by Voronoi Decomposition method is a computational technique used to quantitatively assess ion diffusion pathways by identifying percolation networks, measuring channel widths, and evaluating the accessibility of migration sites within a crystalline material [11]. One of its key advantages is its ability to distinguish conduction dimensionalities, classifying materials based on whether they exhibit one-dimensional (1D), two-dimensional (2D), or three-dimensional (3D) ion transport—a factor that determines long-range ionic conductivity. By mapping the connectivity and spatial distribution of interstitial voids, CAVD identifies the structural constraints that govern ion mobility. CAVD also locates the diffusion bottlenecks that hinder ion migration, which points to the structural modifications most likely to improve transport.

Compared to traditional computational methods such as DFT and MD, CAVD is significantly more computationally efficient, enabling the rapid screening of a large number of materials. While it does not explicitly account for electronic interactions or dynamic effects, it provides a geometric and topological framework for evaluating ionic diffusion characteristics. When integrated with complementary approaches such as BVSE calculations, CAVD offers a more comprehensive understanding of the interplay between crystal structure, interstitial site topology, and ionic transport mechanisms.

**Figure 4:**
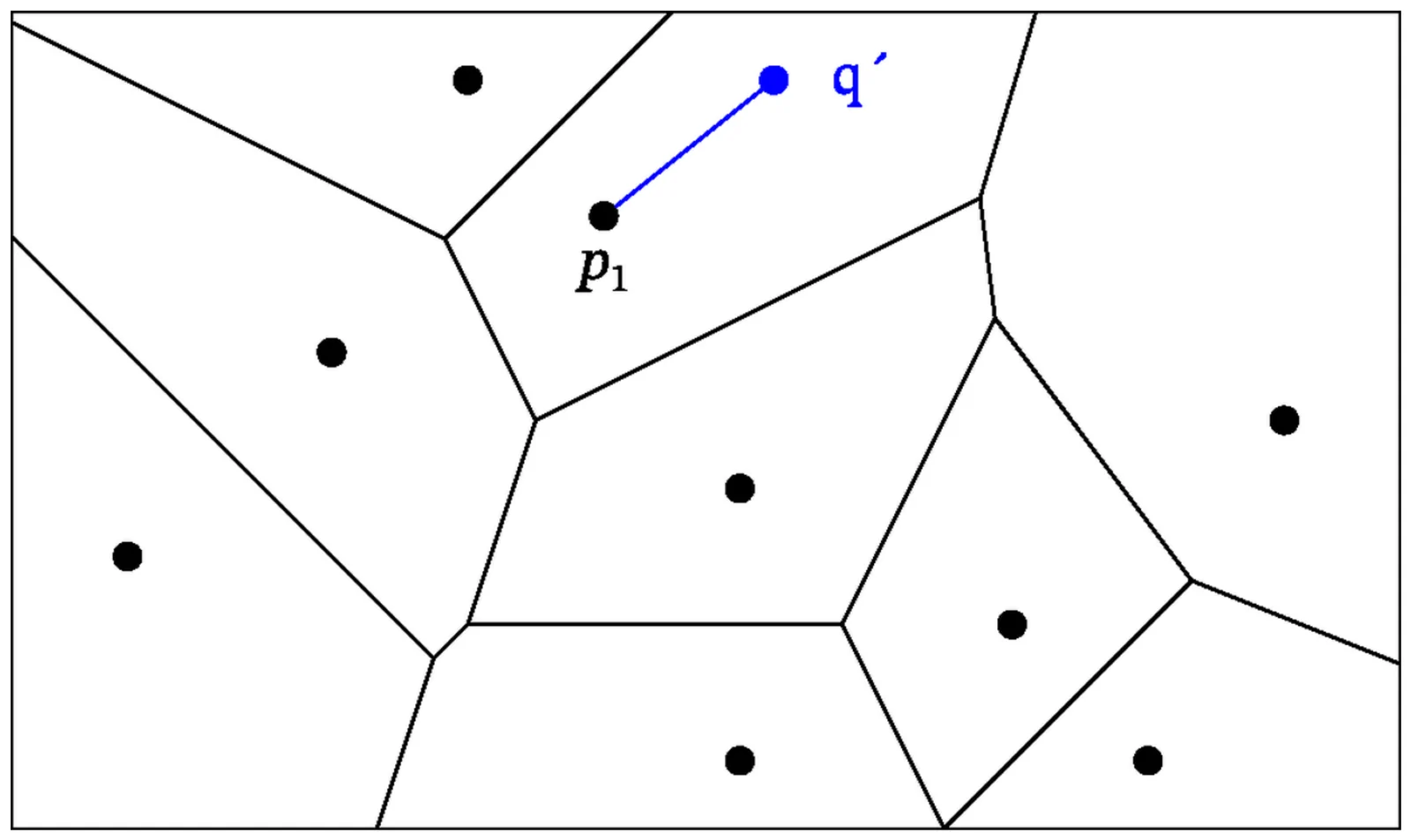
Voronoi decomposition of a set of points in 2D space.

Voronoi decomposition relies on mapping the void space of the crystal structure. In 2D, given a set of points, Voronoi decomposition creates a region (or Voronoi cell) where all points inside that cell are closer to the defining point than to any other point, similarly to the concept of the Wigner–Seitz cell and the Brillouin zone in solid-state physics. For a set of points in the plane, $p_{i}$ where p∈P, we can define the Voronoi diagram Vor(P), illustrated in Figure 4. The Voronoi cell of a point $p_{i}$ is given by

(1) $$V\left(p_{i}\right)=\{x\in R^{2}:|xp_{i}|<|xq|\;\forall \;q\in P\}.$$

This means that the Voronoi cell of $p_{i}$ contains any point *x* that is closer to $p_{i}$ than to any other point q∈P. For instance, consider the point $q^{\prime }$ (marked in blue); since it is within the Voronoi cell of $p_{1}$, there is no other point of the set *P* closer to $q^{\prime }$ than $p_{1}$. Associated with each Voronoi cell are Voronoi edges and Voronoi vertices.

Extending the Voronoi concept to 3D replaces Voronoi cells with Voronoi polyhedra. Using the program Crystal Analysis by Voronoi Decomposition (CAVD) [11], the void space is analyzed and mapped into a Voronoi network. This network consists of interstices (the vertices of the Voronoi polyhedra), which represent the sites between which lithium ions hop. The edges of these polyhedra form channels between interstices, tracing the paths that lithium ions follow. Finally, bottlenecks occur at the narrowest points on the Voronoi edges, defining the limiting size for ionic conduction. In this work, we calculate the interstitial network for all studied polymorphs.

By using CAVD, we can pinpoint the locations where a lithium hop occurs, while BVSE provides insights into the energy landscape of an ion within the crystal. Merging these two methodologies [10,12] enables us to compute the energy along a migration path in a manner similar to NEB calculations, but with reduced computational cost.

Throughout this work we distinguish two dimensionality measures. The energetic dimensionality follows from the BVSE landscape: $E_{n}^{D}$ (n=1,2,3) is the lowest energy at which the Li migration network forms a connected pathway spanning *n* dimensions, and the energetic dimensionality of a structure is the highest *n* that connects at $E_{1}^{D}$. The geometric dimensionality follows from the CAVD void network: the critical channel radius along an axis, $R_{T,x}$ (x=a,b,c), is the radius of the largest spherical probe that can traverse the structure along that axis, and the geometric dimensionality is the number of axes whose critical radius lies within the size window accessible to Li^+^. The two measures are complementary and need not coincide: a geometrically connected network can remain energetically costly in some directions, as in *T*-LiFeO_2_ (geometrically 3D, energetically 1D) and γ-LiFeO_2_ (geometrically 3D, energetically 2D) (Table 2; Figures S3 and S4).

### 2.3. Compound Selection

In this work, the lithium polymorphs Layered, Corrugated Layer, ordered rock salt, and γ-LiFeO_2_ were selected from the Materials Project database, with references mp-19419, mp-757614, mp-755709, and mp-18782. The tetrahedral structure was obtained from the Powder Diffraction File (PDF) database (PDF Card-04-016-2018). The goethite structure was acquired from a DFT-based method capable of resolving atomic positions, since the atomic positions in goethite LiFeO_2_ were previously unresolved [25]. No fully ordered structural model was available for the remaining four polymorphs in Table 1 (α, β, $\beta^{\prime }$, and hollandite). α-LiFeO_2_ is cation-disordered [2,26]. β-LiFeO_2_ is reported as a tetragonal CoO-type cell with four atoms per unit cell (Pearson symbol tI4), which leaves a single cation site shared by Li and Fe [3]. The $\beta^{\prime }$ phase is only partially ordered: neutron refinement gives mixed Fe/Li occupancies on two of its four cation sites [26]. For the hollandite phase, the literature we consulted reports the symmetry of the β-FeOOH parent but no refined atomic coordinates or site occupancies for LiFeO_2_ [2]. BVSE and CAVD analysis requires a defined cation arrangement [12]: for a phase with disordered or mixed site occupancies, any single ordered decoration would probe that particular arrangement rather than the polymorph itself. A proper treatment would require special quasirandom structures or ensemble averaging, which is beyond the scope of this work, so these four phases were not included. Before performing any calculations, each of the six selected cells was converted to a supercell (2×2×2 for all polymorphs except the ordered rock salt, whose starting cell already contains 64 atoms and was expanded to 1×1×2; Table S1 lists all supercell sizes) using Supercell version 2.1.1. The same expansions are reproduced in the deposited workflow using pymatgen version 2023.12.18 [29]. The supercells better represent the migration paths and avoid erroneous results due to atoms being too close to the cell boundaries. Afterwards, all cells were relaxed with chgnet [30]. CHGNet is a graph neural network-based machine-learning interatomic potential pretrained on Materials Project DFT relaxation trajectories. On its held-out test set, it reproduces DFT energies and forces with mean absolute errors of 30 meV atom^−1^ and 77 meVÅ^−1^, respectively [30]. Because CHGNet predicts energies and forces without explicitly solving the electronic-structure problem at every relaxation step, it offers substantially lower computational cost than conventional DFT while retaining near-DFT accuracy. This efficiency enabled the six LiFeO_2_ supercells to be relaxed using a single, consistent computational framework. The relaxation steps and final energies are presented in Figures S1 and S2 in the Supplementary Materials; Figure S2 and Table S1 report the energies normalized per atom, where the six polymorphs span only 22.7 meV atom^−1^. Relaxations used CHGNet 0.3.4 with the FIRE optimizer, simultaneous cell and atomic relaxation, a force threshold of 0.1 eVÅ^−1^, and a maximum of 500 steps. The transport analysis then applied identical settings to all six structures: BVSE energy landscapes for Li^+^ on a 0.1 Å grid, and CAVD channel screening with per-phase lower radius bounds of 0.48–0.53 Å and an upper bound of 1.0 Å. The complete parameter set, with all inputs and outputs, is preserved in the deposited analysis code (see Data Availability Statement).

## 3. Results and Discussion

The results presented in this section were obtained using the combination of Bond Valence Site Energy (BVSE) calculations and Crystal Analysis by Voronoi Decomposition (CAVD) [10,12] described in Section 2. This integrated approach provides a combined understanding of the energetic and geometric constraints governing ion transport in the six polymorphs studied. Table 2 summarizes the calculated energy thresholds, dimensionalities, and critical channel radii for all six polymorphs.

### 3.1. BVSE Migration Paths for LiFeO_2_ Polymorphs

#### 3.1.1. Layer and Corrugated Layer Structures

**Figure 5:**
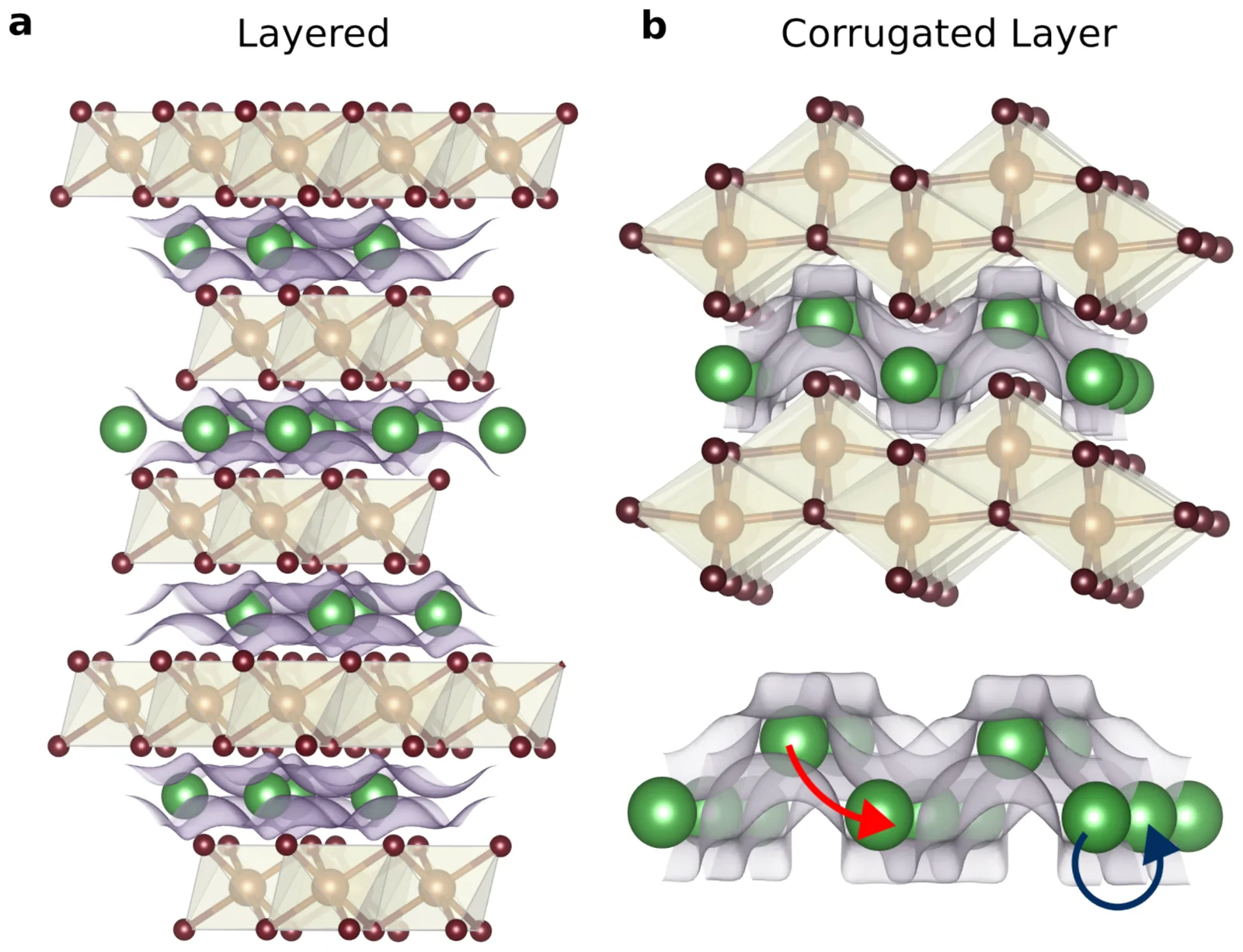
Li-ion migration pathways in LiFeO_2_ polymorphs obtained using the BVSE method: (**a**) layered and (**b**) corrugated layer structures. In (**b**), the blue arrow marks the straight migration path parallel to the *a*-axis and the red arrow the zig-zag route.

In the layered LiFeO_2_ polymorph, BVSE analysis yields energy barriers of 1.17 eV for both 1D and 2D diffusion, as well as a substantially higher value of 10.47 eV for 3D diffusion. The high structural symmetry and regular arrangement of FeO_6_ layers and Li ions promote efficient packing and lower in-plane barriers, so that the energy requirements are similar along the *a* and *b* directions. As shown in Figure 5a, such symmetry facilitates straightforward Li-ion migration pathways, supporting good in-plane ionic conductivity. In contrast, the corrugated layer structure (Figure 5b) exhibits higher and more anisotropic barriers: 1.21 eV for 1D, 1.36 eV for 2D, and 9.73 eV for 3D conduction. Orthorhombic distortions lead to variations in Fe−O bond lengths and angles, influencing layer spacing and the geometry of interlayer regions [19]. As a result, the corrugated layer supports a quasi-2D conduction pathway with slightly higher barriers compared to the layered structure. Migration along a straight path parallel to the $\vec{a}$-axis (blue arrow) is more favorable than the zig-zag route (red arrow), whereas 3D migration remains energetically costly.

#### 3.1.2. Tetrahedral and Goethite Structures

**Figure 6:**
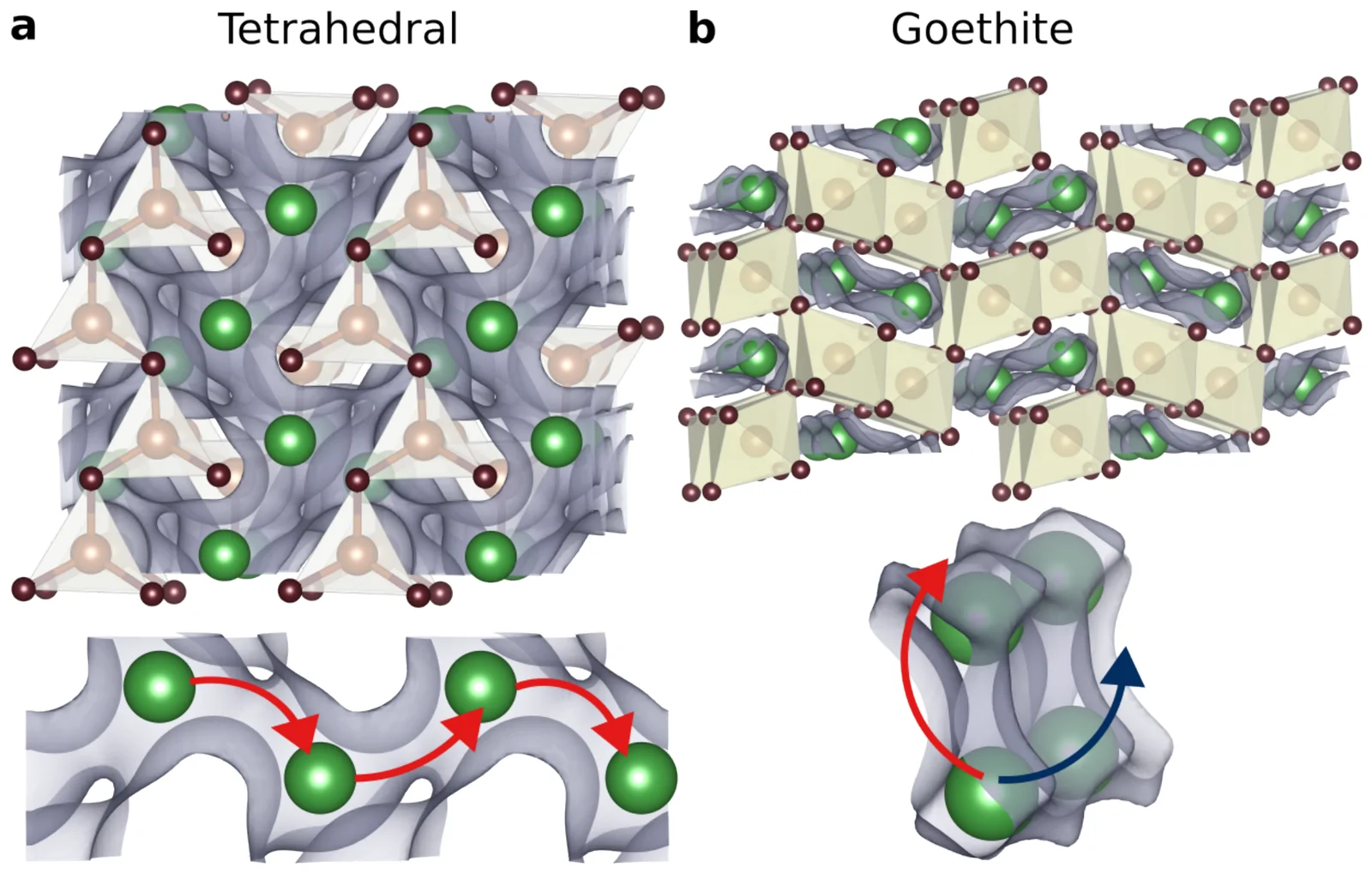
Li-ion migration pathways in LiFeO_2_ polymorphs obtained using the BVSE method: (**a**) tetrahedral and (**b**) goethite structures. In (**b**), the blue arrow indicates the favorable one-dimensional migration axis and the red arrow the restricted out-of-plane direction.

As discussed in Section 1.1.2, *T*-LiFeO_2_ was hypothesized to enable multi-dimensional conduction. BVSE calculations confirm this by revealing energy barriers of 0.70 eV for 1D diffusion, and 1.23 eV for both 2D and 3D diffusion. The easiest migration is therefore one-dimensional, and the network connects in all three directions only at the higher threshold. That threshold is still the second lowest three-dimensional value of the six polymorphs, behind only the ordered rock salt phase, so conduction in all three directions remains far more accessible in *T*-LiFeO_2_ than in the layered, corrugated, or goethite structures (Figure 6a).

For goethite LiFeO_2_, the BVSE results are 0.77 eV for 1D diffusion and significantly higher at 3.50 eV for both the 2D and 3D directions. This indicates that the goethite structure strongly favors Li-ion migration along a single axis (blue arrow), with structural distortions and less accessible interlayer regions limiting out-of-plane conductivity (red arrow; Figure 6b).

#### 3.1.3. γ-LiFeO_2_ and Ordered Rock Salt Structures

**Figure 7:**
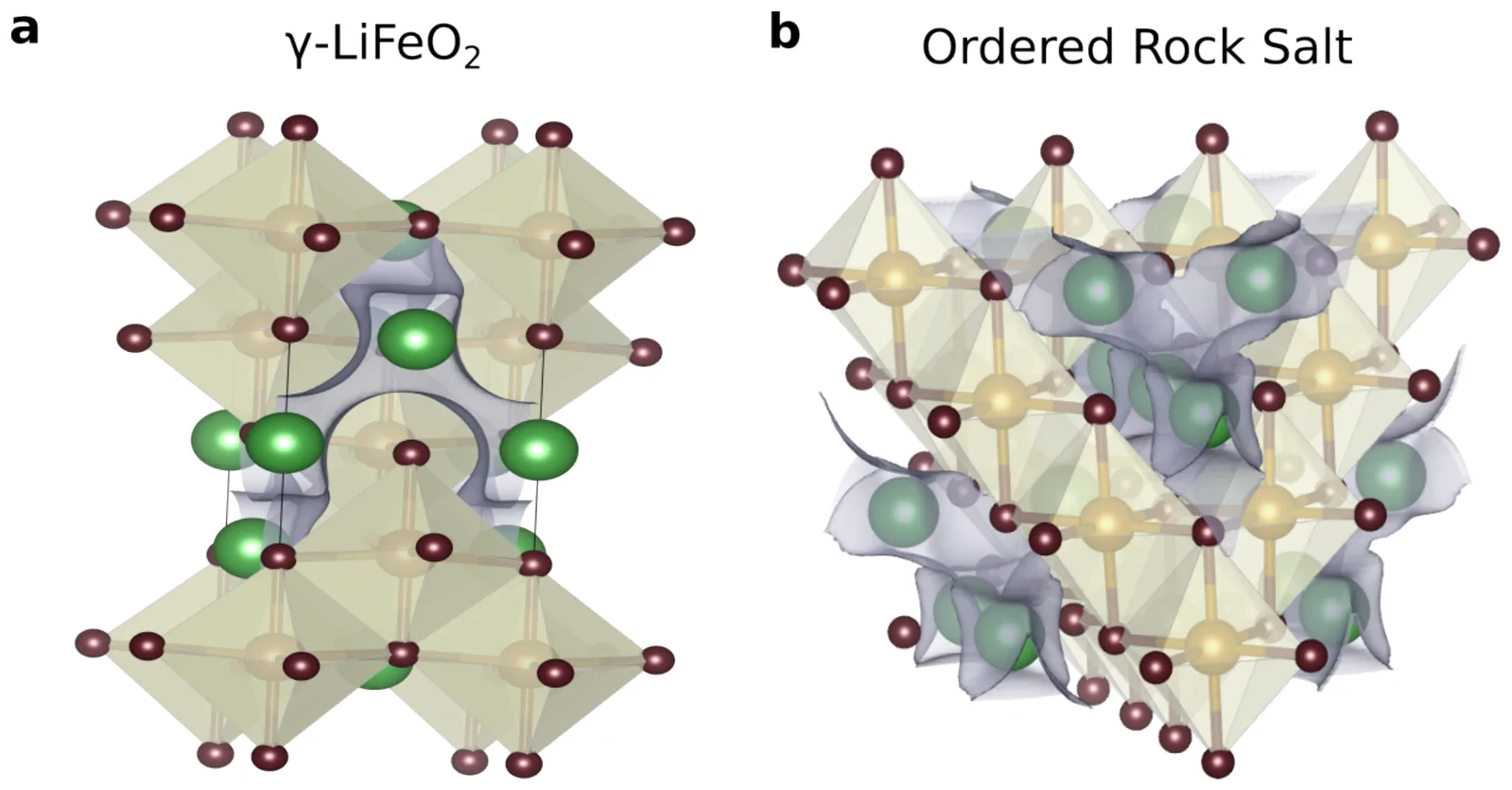
Li-ion migration pathways in LiFeO_2_ polymorphs obtained using the BVSE method: (**a**) γ-LiFeO_2_ and (**b**) ordered rock salt.

γ-LiFeO_2_ (Figure 7a) offers the least accessible transport of the six polymorphs: even its lowest-barrier migration direction requires 1.67 eV (1D and 2D diffusion, with 3D at 1.69 eV), more than the easiest migration direction of any other polymorph, consistent with the findings from the literature. Urban et al. demonstrated that γ-LiFeO_2_ possesses only two-transition-metal (2-TM) channels, which generally remain inactive due to strong electrostatic repulsion in the tetrahedral sites [17]. This high energy barrier severely limits Li-ion mobility, resulting in poor ionic conductivity and constraining its suitability for battery applications. Belak et al. further corroborated this observation in their work on lithiated anatase (LiTiO_2_), reporting similarly high energy migration barriers [31].

Bond valence calculations also confirm that γ-LiFeO_2_ is a poor ionic conductor, exhibiting high, nearly isotropic, barriers of 1.67 eV to 1.69 eV. This finding aligns well with the earlier discussion and highlights the challenge of achieving efficient Li-ion transport in this polymorph.

In contrast, the ordered rock salt polymorph (Figure 7b) stands out with notably low and isotropic energy barriers of 0.41 eV. Its well-ordered cationic arrangement and expanded interstitial pathways enable rapid, multidirectional Li-ion migration. This structural attribute is consistent with the improved electrochemical capacity reported when phase transformations lead to the formation of this polymorph [15,18]. Within this transport comparison, the ordered rock salt phase is the most favorable of the six polymorphs.

Overall, BVSE analysis links crystal structure directly to Li-ion mobility in LiFeO_2_. The ordered rock salt and tetrahedral structures support relatively easy ion migration in one or more directions, potentially enhancing ionic conductivity. By contrast, corrugated, goethite, and especially γ-LiFeO_2_ forms impose higher barriers, restricting Li-ion transport. Our estimations highlight that deliberate manipulation of crystal structure is key to optimizing transport. Figure S3 in the Supplementary Materials presents a summary of the BVSE energy thresholds ($E_{1}^{D}$) and the resulting energetic dimensionality for each polymorph. Additionally, animated 3D visualizations of the migration pathway isosurfaces for all polymorphs are provided as a single Supplementary Video (Video S1).

### 3.2. Joint CAVD–BVSE Analysis

#### 3.2.1. Layer and Corrugated Layer Structures

**Figure 8:**
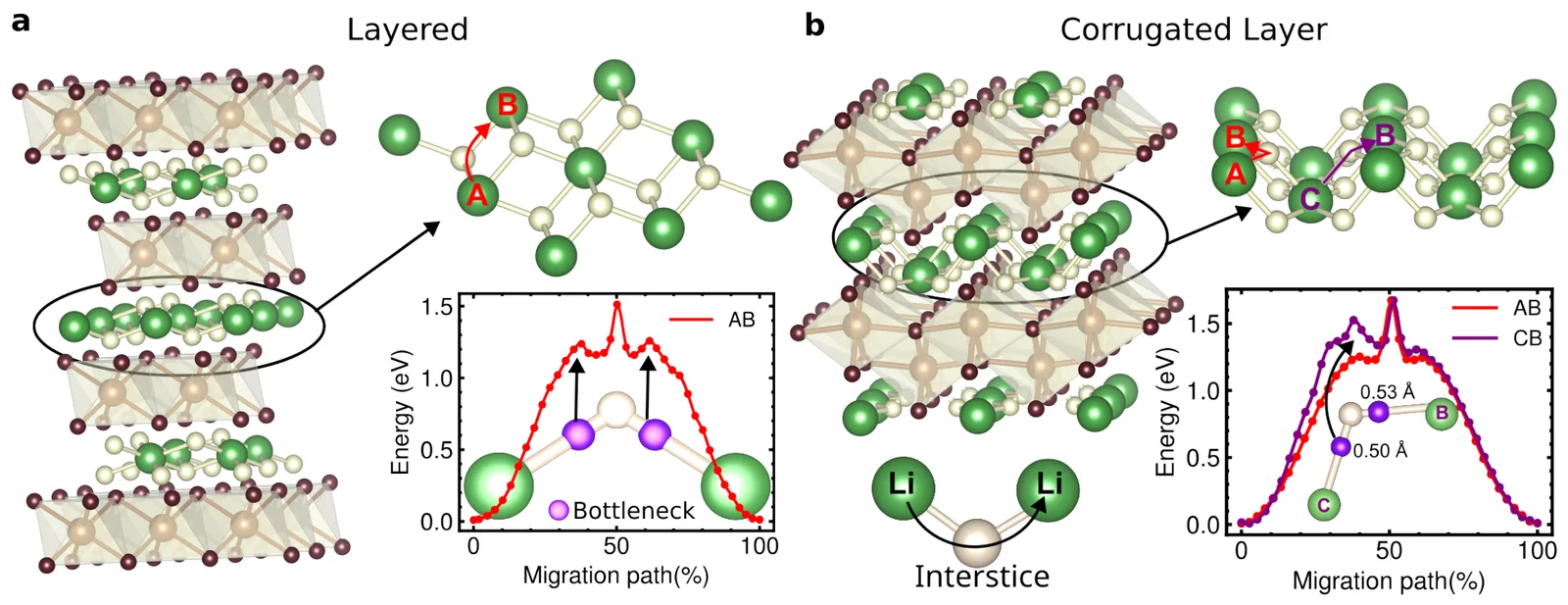
Interstitial network and energy migration paths for LiFeO_2_ polymorphs obtained using the joint method of CAVD and BVSE: (**a**) layered LiFeO_2_ polymorph and (**b**) corrugated layer LiFeO_2_ polymorph. The interstitial networks are shown alongside the energy profiles for unique migration paths, highlighting the critical radii and bottlenecks that influence Li-ion mobility.

Figure 8a,b presents the interstitial networks from CAVD analysis, alongside BVSE-derived energy profiles for the fully lithiated layered and corrugated layer LiFeO_2_ polymorphs. CAVD identifies the interstitial network and its accessibility, complementing the BVSE energy landscape. For the layered LiFeO_2_, CAVD results confirm a well-connected 2D network, with critical radii ($R_{T}$) of 0.52 Å, 0.52 Å, and 0.21 Å along $\vec{a}$, $\vec{b}$, and $\vec{c}$, respectively. These $R_{T}$ values align with the BVSE findings, indicating that Li ions can move easily in-plane. Energy profiles peak between 40% and 60% of the migration path length, corresponding to bottlenecks where the interstitial volume narrows.

For the corrugated layer polymorph, the CAVD analysis yields $R_{T}$ values of 0.53 Å, 0.50 Å, and 0.23 Å along $\vec{a}$, $\vec{b}$, and $\vec{c}$, again in agreement with BVSE data. The favorable conduction direction is along $\vec{a}$ (path labeled AB in Figure 8b), while migration within the lithium bilayer (path labeled CB in Figure 8b) encounters narrower bottlenecks. For the narrower 0.50 Å bottleneck associated with the bilayer path (CB), the closest FeO_6_-octahedra oxygen atom is only 1.76 Å away, whereas the slightly larger 0.53 Å intra-layer bottleneck maintains a longer 1.79 Å oxygen–ion distance. These differences in bottleneck size and proximity to oxygen atoms correlate with the higher migration energies observed.

#### 3.2.2. Tetrahedral and Goethite Structures

**Figure 9:**
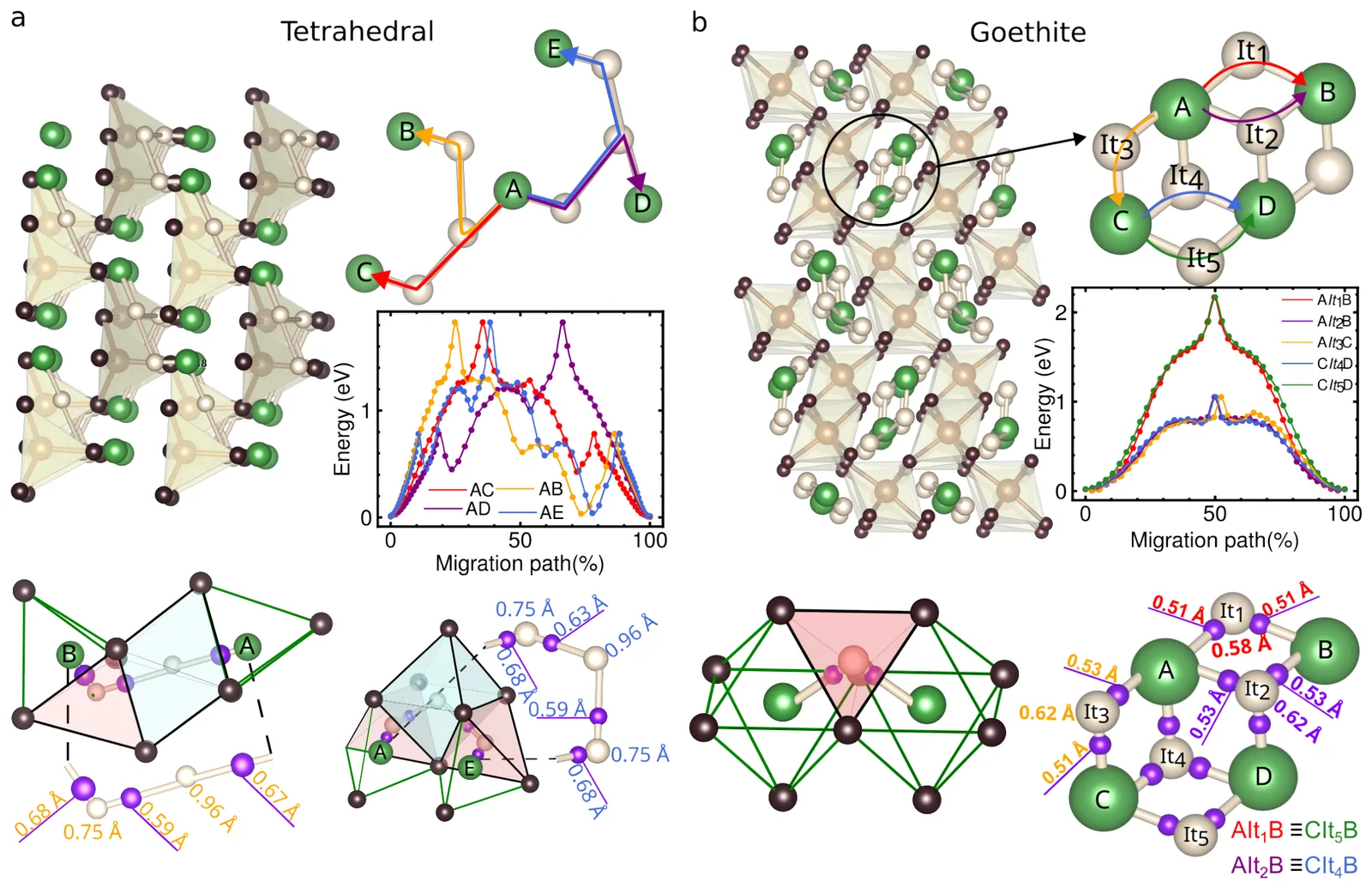
Interstitial network and energy migration paths for LiFeO_2_ polymorphs obtained using the joint method of CAVD and BVSE: (**a**) tetrahedral LiFeO_2_ polymorph and (**b**) goethite LiFeO_2_ polymorph. The interstitial networks are shown alongside the energy profiles for unique migration paths, highlighting the critical radii and bottlenecks that influence Li-ion mobility. In (**a**), the octahedral and tetrahedral interstices are colored blue and red, respectively, and arrows denote the migration paths AB (yellow), AC (red), AD (purple), and AE (blue). In (**b**), arrows denote the paths A–It_1_–B (red), A–It_2_–B (purple), C–It_4_–D (blue), C–It_5_–D (green), and A–It_3_–C (yellow).

*T*-LiFeO_2_, whose interstitial network is shown in Figure 9a, exhibits $R_{T}$ values of 0.64 Å along all three crystallographic directions. These values are consistent with the BVSE results, the critical radius being essentially isotropic across the three axes.

If we focus on migration paths around a particular lithium atom (labeled A), we find that Li can move along either two-interstice paths (e.g., paths AB (yellow arrow), AC (red arrow), and AD (purple arrow)) or three-interstice paths (e.g., path AE (blue arrow)). The two-interstice paths follow a t–o–t–t sequence, meaning the migration starts and ends at a tetrahedral site, passing through an octahedral and then another tetrahedral interstitial site. Based on the interstice and bottleneck sizes, one might initially expect that the path passing through the octahedral interstice (painted in blue on the left corner of Figure 9a) along AB would have a lower migration energy than paths passing through the tetrahedral site. However, this is not the case.

The explanation lies in the local environment: the octahedral interstice is constrained by four bottlenecks, while the tetrahedral interstice (painted in red) is constrained by only three bottlenecks. Consequently, the two-interstice migration path actually shows higher migration energy when passing through the octahedral interstice. The three-interstice path (t–t–o–t–t) also follows a similar trend, showing a lower energy barrier when passing through the tetrahedral site and reaching its highest energy at the octahedral interstitial site.

Regarding the goethite interstitial network shown in Figure 9b, the $R_{T}$ values are 0.44 Å, 0.53 Å, and 0.44 Å along the $\vec{a}$, $\vec{b}$, and $\vec{c}$ directions, respectively. These values align with the 1D conduction behavior predicted by BVSE calculations. In the goethite polymorph, all identified migration paths follow an o–t–o (octahedral–tetrahedral–octahedral) configuration.

To better understand these migration pathways, we can draw an analogy to a corrugated layer structure composed of lithium bilayers. In this model, the upper layer contains atoms A and B, and the lower layer contains atoms C and D. Within each layer, there are two unique migration paths. For example, in the upper layer, lithium can move from A to B via two possible interstitial sites (It_n_), forming paths A–It_1_–B (red arrow) and A–It_2_–B. Among these, the A–It_2_–B (purple arrow) path is more energetically favorable. In the lower layer, the analogous paths C–It_4_–D (blue arrow) and C–It_5_–D (green arrow) mirror those in the upper layer, but here C–It_4_–D emerges as the more favorable route. This preferred path is located on the opposite side of the layer compared to the favored route in the upper layer, which reflects the asymmetry of the energetic landscape.

A third unique path enables the transition between the upper and lower layers (A–It_3_–C) (yellow arrow). Despite involving a slightly smaller bottleneck, this vertical transition remains energetically accessible, showing only a minor increase in the energy barrier at around 60% of the path length, which corresponds to the passage through the bottleneck.

Overall, these findings highlight that while goethite LiFeO_2_ fundamentally supports 1D conduction, the exact migration routes within and between layers are highly dependent on local interstitial environments and bottleneck sizes. These local differences produce measurable variations in the energy barriers along the different routes.

#### 3.2.3. γ-LiFeO_2_ and Ordered Rock Salt Structures

**Figure 10:**
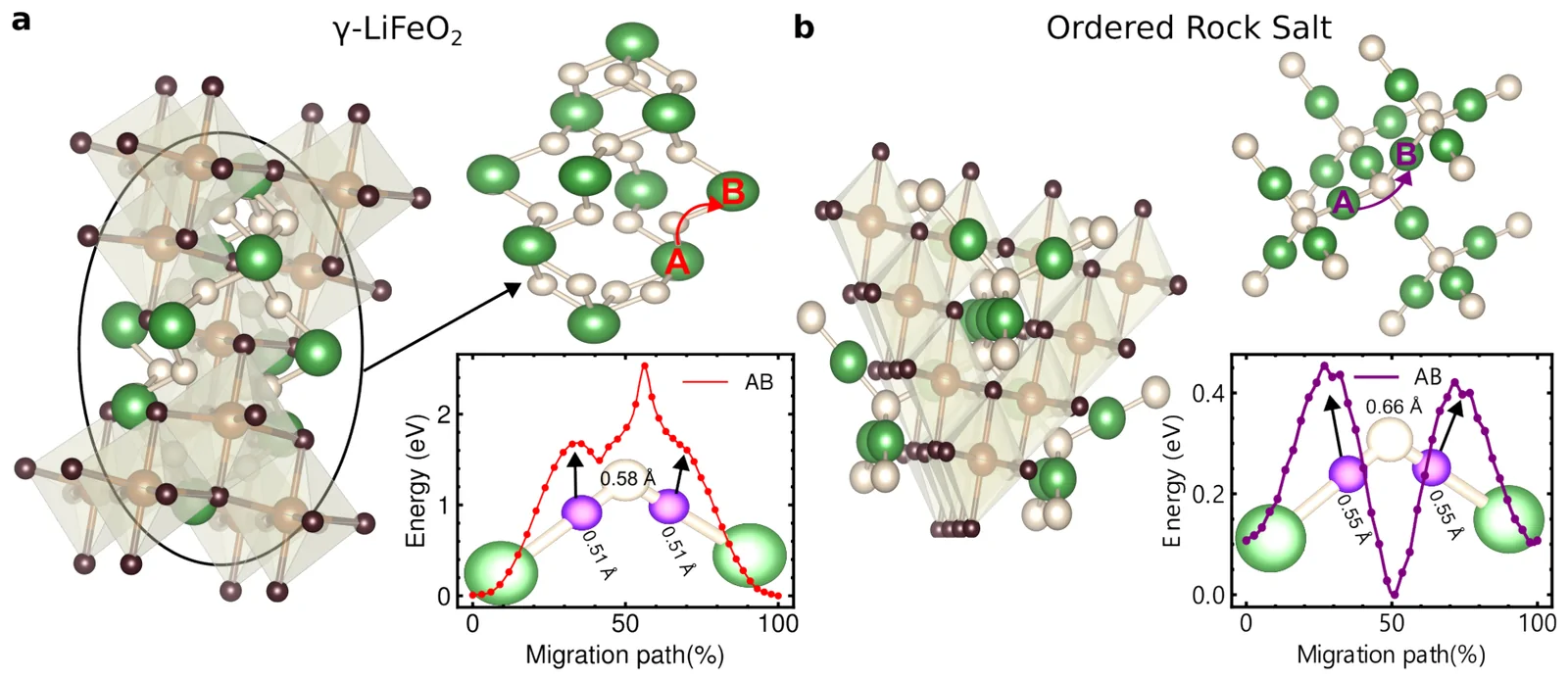
Interstitial network and energy migration paths for LiFeO_2_ polymorphs obtained using the joint method of CAVD and BVSE: (**a**) γ-LiFeO_2_ polymorph and (**b**) ordered rock salt LiFeO_2_ polymorph. The interstitial networks are shown alongside the energy profiles for unique migration paths, highlighting the critical radii and bottlenecks that influence Li-ion mobility.

γ-LiFeO_2_ exhibits $R_{T}$ values of 0.51 Å, 0.51 Å, and 0.51 Å along each crystallographic direction. These uniformly low values make the void network geometrically 3D, in line with the nearly isotropic BVSE barriers, and their small size is consistent with the high migration energy barriers. As shown in Figure 10a, only one unique migration path is identified. The small interstitial spaces and narrow bottlenecks along this o–t–o pathway impose substantial energetic penalties on ion migration, which aligns with the high barriers reported in the literature, as discussed in Section 1.1.3.

In contrast, the ordered rock salt LiFeO_2_ polymorph displays $R_{T}$ values of 0.55 Å, 0.55 Å, and 0.55 Å, supporting both the 3D conduction and the low energy barriers anticipated by BVSE results. Similar to the γ phase, only one unique o–t–o path is observed. However, in this polymorph the interstitial and bottleneck dimensions are larger, effectively reducing the Li-ion migration barrier to a very low value of 0.41 eV. This finding contrasts with the other five polymorphs and shows how the openness of the lattice controls ionic mobility.

Critical channel radius $R_{T}$ values with corresponding geometric dimensionality (1D, 2D, or 3D) are available in Figure S4, which plots the largest of the three axial values, $\max\{R_{Ta},R_{Tb},R_{Tc}\}$, rather than the per-axis values listed in Table 2, and an overall plot comparing conduction descriptors (CAVD-derived $R_{T}$ values versus BVSE energy thresholds $E_{1}^{D}$) is available in Figure S5.

## 4. Conclusions

This study compares Li-ion transport side by side across six LiFeO_2_ polymorphs: layered, corrugated, tetrahedral, goethite, γ, and ordered rock salt. Bond Valence Site Energy (BVSE) analysis and Crystal Analysis by Voronoi Decomposition (CAVD) are both established methods, and we applied them under a single protocol so that diffusion pathways, energy thresholds, and conduction dimensionalities can be compared directly across all six structures.

The six polymorphs fall into a hierarchy of transport behavior (Table 2). (i) The most favorable is the ordered rock salt structure, the only polymorph for which geometry and energetics agree on kinetically accessible three-dimensional transport: its void network is geometrically 3D and its migration network connects along all three axes at the lowest energy threshold of the set (0.41 eV). (ii) Tetrahedral LiFeO_2_ has the following structure: its void network is also geometrically 3D, and one migration direction opens at the second-lowest energy threshold of the set (0.70 eV), but full three-dimensional connectivity costs three times the rock salt value (1.23 eV). (iii) The layered and corrugated structures conduct within their layer planes at intermediate energy thresholds, while out-of-plane migration lies roughly an order of magnitude higher, so the transport remains quasi-two-dimensional. (iv) The most constrained are the γ and goethite structures, where transport fails in two different ways. In the γ phase, geometry and energetics disagree: the void network is geometrically 3D and nearly isotropic, yet even its easiest migration direction costs more than full three-dimensional connectivity in the tetrahedral or rock salt structures, so the network is kinetically ineffective. The disagreement, however, is confined to the dimensionality labels: the γ channels are the narrowest of the three geometrically 3D polymorphs, so even the geometric analysis points to marginal accessibility. In goethite, geometry and energetics agree on strongly anisotropic transport: Li ions migrate along a single axis at low energy, but a network spanning two or three dimensions connects only at several times that energy.

Effective Li-ion transport in these polymorphs is therefore governed by the combined effects of network connectivity, bottleneck geometry, and local interstitial energetics, rather than by the nominal dimensionality of the diffusion network alone. Small differences in local distortions, bottleneck sizes, and interstice dimensions are enough to decide whether a geometrically connected network is kinetically accessible. Crystal–chemical or microstructural strategies that enlarge the narrow bottlenecks and interstices are a promising route to improving Li-ion transport in LiFeO_2_-based cathodes.

The hierarchy presented above reflects ionic transport descriptors only. The migration-energy thresholds and critical channel radii obtained from the BVSE/CAVD analysis are approximate descriptors of Li-ion transport and should not be interpreted as experimentally measured ionic conductivity or battery performance. Although these methods have been shown to reproduce trends in migration pathways and energy barriers across related structures [13] and to exhibit semi-quantitative agreement with first-principles calculations and available experimental data [10,12], a connected low-energy migration network is a necessary, but not sufficient, condition for high ionic conductivity [10,11]. Layered LiFeO_2_ has been reported to transform toward an ordered rock salt phase on cycling, with an accompanying capacity increase [15,18]. The phase has so far been observed only within mixed-phase samples and has not been synthesized in phase-pure form. Whether it can function as a practical cathode also depends on its thermodynamic accessibility, electrochemical stability, electronic conductivity, and reversibility during cycling. These properties were not evaluated in this work and remain targets for follow-up study, together with the direct verification of the 0.41 eV rock salt threshold by DFT-NEB and by transport measurements on phase-pure material.

## Figures and Tables

**Table 2:** Calculated Li-ion transport descriptors for the six cation-ordered LiFeO_2_ polymorphs. $E_{n}^{D}$ is the lowest energy at which a connected Li migration network spans *n* dimensions, and the energetic dimensionality is the highest dimensionality that connects at $E_{1}^{D}$ (Figure S3). $R_{T,a}$, $R_{T,b}$, and $R_{T,c}$ are the critical channel radii along the three crystallographic axes, and the geometric dimensionality is the number of axes whose critical radius lies within the Li thresholds (Figure S4).

Polymorph	$E_{1}^{D}$	$E_{2}^{D}$	$E_{3}^{D}$	Energ.	$R_{T,a}$	$R_{T,b}$	$R_{T,c}$	Geom.
	(eV)	(eV)	(eV)	dim	(Å)	(Å)	(Å)	dim
Layered	1.17	1.17	10.47	2D	0.52	0.52	0.21	2D
Corrugated layer	1.21	1.36	9.73	1D	0.53	0.50	0.23	2D
*T*-LiFeO_2_	0.70	1.23	1.23	1D	0.64	0.64	0.64	3D
Goethite	0.77	3.50	3.50	1D	0.44	0.53	0.44	1D
γ	1.67	1.67	1.69	2D	0.51	0.51	0.51	3D
Ordered rock salt	0.41	0.41	0.41	3D	0.55	0.55	0.55	3D

## Data Availability

The crystal structures, numerical results, BVSE energy grids, migration-path data, and analysis code supporting this study are openly available in the Zenodo repository at https://doi.org/10.5281/zenodo.21648625. The crystal structures analyzed in this work were obtained from the Materials Project database (entries mp-19419, mp-757614, mp-755709, and mp-18782) and the Powder Diffraction File database (PDF Card-04-016-2018).

## Supplementary Materials

The following supporting information can be downloaded at https://www.mdpi.com/article/10.3390/ma19163378/s1. Figure S1: Convergence behavior of the LiFeO_2_ polymorphs during structural optimization. Relative energy (left axis) and atomic forces (right axis) versus optimization step for each polymorph’s supercell, calculated with CHGNet; Figure S2: Comparison of the final relaxation energies per atom for the six LiFeO_2_ polymorphs after full structural relaxation with CHGNet; Table S1 lists the corresponding total energies and supercell sizes; Table S1: Supercell composition and CHGNet final energies for the six cation-ordered LiFeO_2_ polymorphs, ordered by energy per atom; Figure S3: Bond-Valence Site Energy (BVSE) energy-threshold analysis of the six polymorphs. Each bar is ($E_{1}^{D}$), the lowest energy at which a connected Li migration network spans one dimension, and the label gives the highest dimensionality that connects at that same energy. This is an energetic criterion, and it does not always match the geometric dimensionality of Figure S4 (Section 2.2 of the main text); Figure S4: Geometric conduction characteristics from CAVD analysis. $R_{T}$ is the critical channel radius, plotted here as the largest of the three per-axis values, max{$R_{\mathrm{Ta}}$, $R_{\mathrm{Tb}}$, $R_{\mathrm{Tc}}$} (Table 2 of the main text), and the label gives the geometric dimensionality, the number of crystallographic axes whose channel radius lies within the Li thresholds (Section 2.2 of the main text). It says where the void network is wide enough and connected, not whether migration along those axes is energetically accessible. Compare the energetic dimensionalities in Figure S3; Figure S5: Comparison between the two conduction descriptors: CAVD critical channel radii ($R_{T}$) against the BVSE energy thresholds $E_{1}^{D}$ of Figure S3; Video S1: Animated 3D visualizations of the Li-ion migration pathway isosurfaces, provided as a single multimedia file showing each LiFeO_2_ polymorph in sequence: layered, corrugated layer, tetrahedral (*T*-LiFeO_2_), goethite, γ-LiFeO_2_, and ordered rock salt.

## Abbreviations

The following abbreviations are used in this manuscript:

LIB	Lithium-ion battery
BVSE	Bond Valence Site Energy
CAVD	Crystal Analysis by Voronoi Decomposition
MEP	Minimum-energy path
DFT	Density functional theory
NEB	Nudged elastic band
MD	Molecular dynamics
TM	Transition metal
SAED	Selected-area electron diffraction
$R_{T}$	Critical channel radius
$E_{a}$	Activation energy
