# Supplementary material for: Understanding Ionic Transport in LiFeO2 Polymorphs: Pathways to Enhancing Lithium-Ion Battery Performance
Source: Materials (Basel). 2026 Aug 8;19(16):3378. doi: 10.3390/ma19163378 (PMC13514424; doi:10.3390/ma19163378)
Supplement: Supplementary file 1 [file materials-19-03378-s001.zip › materials-4431084-supplementary.pdf]

Supplementary Materials: Understanding Ionic Transport in $LiFeO_2$ Polymorphs: Pathways to Enhancing Lithium-Ion Battery Performance

João R. Da Fonseca, Borja Caja-Muñoz, María E. Dávila, Juan P. Martínez-Pastor, Juan F. Sánchez-Royo and Maria C. Asensio

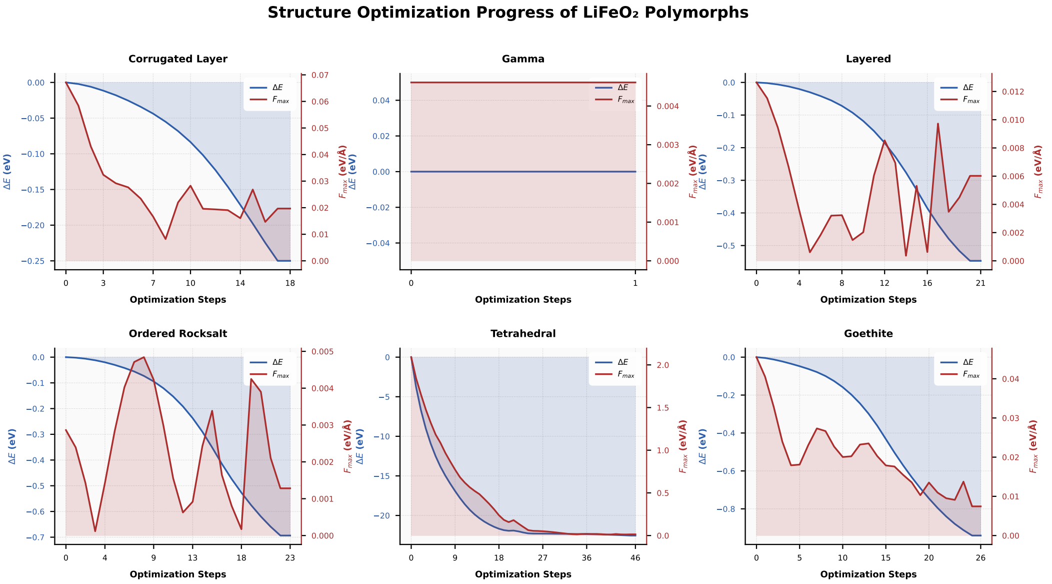

Figure S1. Convergence behavior of the $LiFeO_2$ polymorphs during structural optimization. Relative energy (left axis) and atomic forces (right axis) versus optimization step for each polymorph's supercell, calculated with CHGNet.

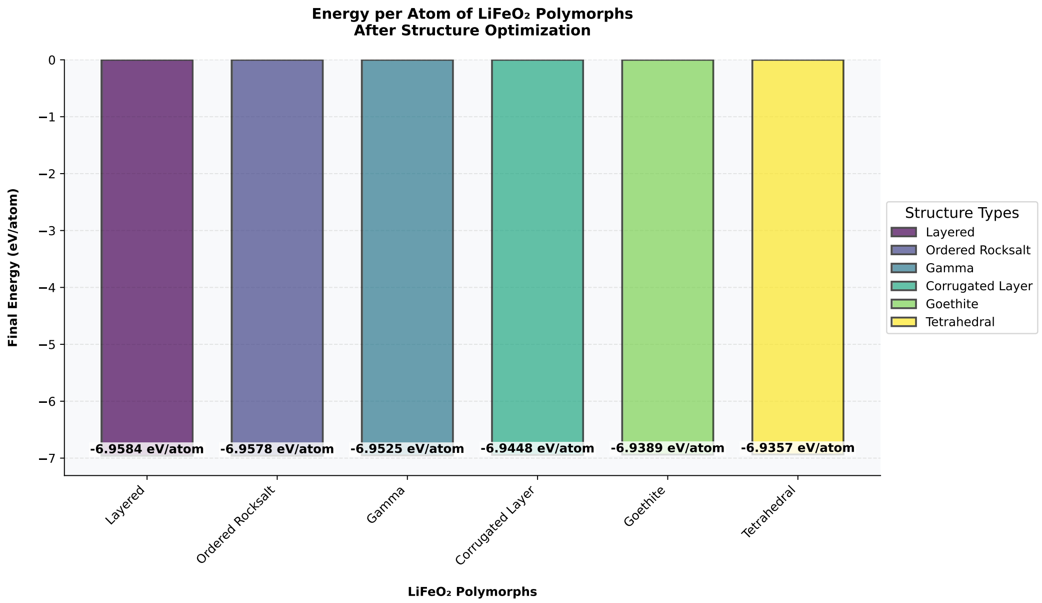

Figure S2. Comparison of the final relaxation energies per atom for the six $LiFeO_2$ polymorphs after full structural relaxation with CHGNet; Table S1 lists the corresponding total energies and supercell sizes.

Table S1. Supercell composition and CHGNet final energies for the six cation-ordered $LiFeO_2$ polymorphs, ordered by energy per atom.

Polymorph	Supercell	Atoms	E_{final} (eV)	E_{final} (eV atom^{-1})
Layered	$2 \times 2 \times 2$	96	−668.007	−6.9584
Ordered rock salt	$1 \times 1 \times 2$	128	−890.593	−6.9578
γ	$2 \times 2 \times 2$	128	−889.924	−6.9525
Corrugated layer	$2 \times 2 \times 2$	64	−444.466	−6.9448
Goethite	$2 \times 2 \times 2$	128	−888.184	−6.9389
T-$LiFeO_2$	$2 \times 2 \times 2$	128	−887.770	−6.9357

All six supercells are stoichiometric $LiFeO_2$, with four atoms per formula unit. Accordingly, the energies per formula unit are four times the values reported in eV atom^{-1}. After normalization, the six phases span only 22.7 meV atom^{-1}, which is below the 30 meV atom^{-1} energy mean absolute error reported for CHGNet (Section 2.3 of the main text). The resulting ordering therefore does not resolve the relative stability of the six polymorphs.

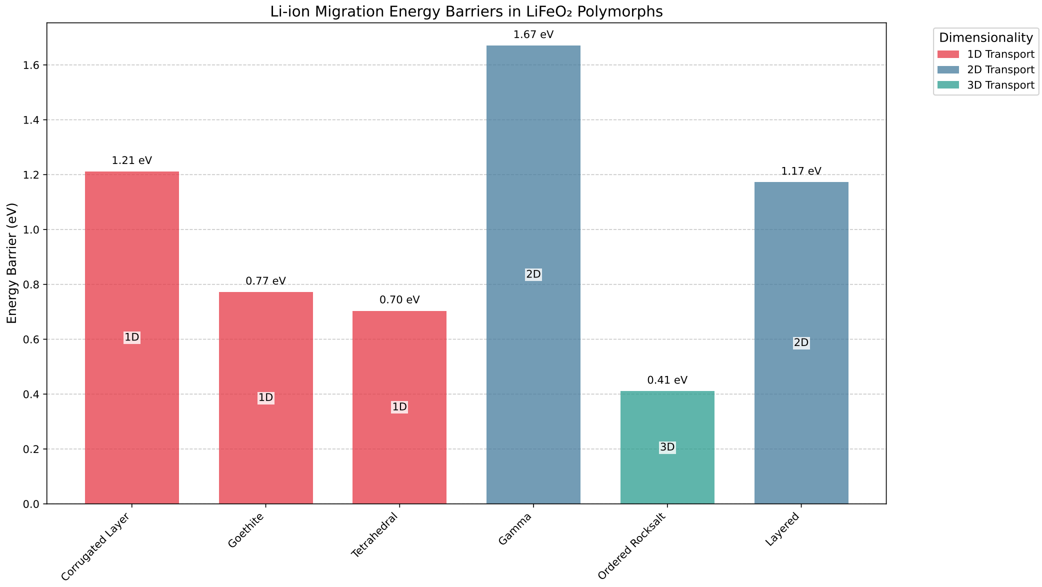

Figure S3. Bond-Valence Site Energy (BVSE) energy-threshold analysis of the six polymorphs. Each bar is E_1^D, the lowest energy at which a connected Li migration network spans one dimension, and the label gives the highest dimensionality that connects at that same energy. This is an energetic criterion, and it does not always match the geometric dimensionality of Figure S4 (Section 2.2 of the main text).

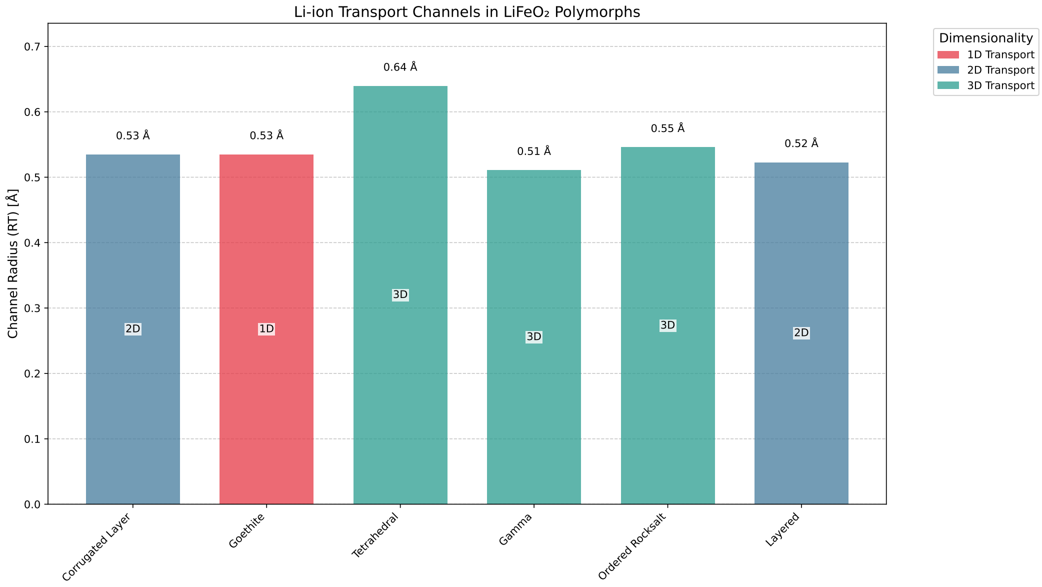

Figure S4. Geometric conduction characteristics from CAVD analysis. R_T is the critical channel radius, plotted here as the largest of the three per-axis values, $\max\{R_{Ta}, R_{Tb}, R_{Tc}\}$ (Table 2 of the main text), and the label gives the geometric dimensionality, the number of crystallographic axes whose channel radius lies within the Li thresholds (Section 2.2 of the main text). It says where the void network is wide enough and connected, not whether migration along those axes is energetically accessible. Compare the energetic dimensionalities in Figure S3.

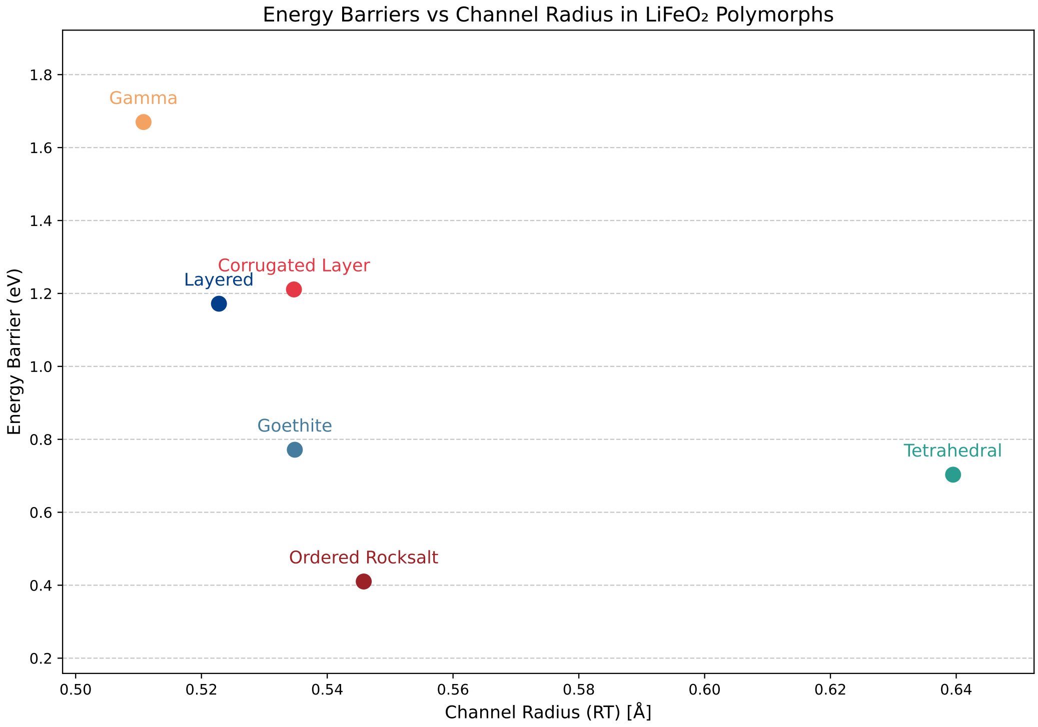

Figure S5. Comparison between the two conduction descriptors: CAVD critical channel radii (R_T) against the BVSE energy thresholds E_1^D of Figure S3.

Supplementary Video

Video S1: animated 3D visualizations of the Li-ion migration pathway isosurfaces, provided as a single multimedia file showing each $LiFeO_2$ polymorph in sequence: layered, corrugated layer, tetrahedral (T-$LiFeO_2$), goethite, γ-$LiFeO_2$, and ordered rock salt.

https://doi.org/10.3390/ma1010000
